# Secondary Trauma by Internet Content Moderation: a Case Report

**DOI:** 10.1192/j.eurpsy.2024.1383

**Published:** 2024-08-27

**Authors:** L. Martinez-Sadurni, F. Casanovas, C. Llimona, D. Garcia, R. Rodriguez-Seoane, J. I. Castro

**Affiliations:** Institut de Neuropsiquiatria i Addiccions (INAD), Hospital del Mar, Barcelona, Spain

## Abstract

**Introduction:**

In recent years, a global debate has emerged regarding the protection of Internet users from exposure to harmful content. Content moderation is defined as the organized practice of filtering user-generated content posted on internet, social networks, and media to determine the appropriateness of the content for a site, locality, or jurisdiction. The growing volume of this content along with the psychological impact of this activity have promoted the application of automated approaches based on artificial intelligence and machine-learning. However, the changing characteristics of content, as well as the cultural differences that influence its appropriateness, mean that human moderation of Internet content currently continues to exist. Psychological effects of this activity such as symptoms of post-traumatic stress disorder (PTSD) could represent an example of secondary trauma.

**Objectives:**

Our aim is to describe a clinical case of post-traumatic stress disorder presenting with specific traumatic exposure idiosyncrasy that could lead to a better consequence characterization of a recent social phenomena such as internet content moderation.

**Methods:**

We expose the clinical case of a woman with emotional distress who was reffered to our outpatient psychiatric unit in Barcelona in 2022 after five years working as an internet content moderator.

**Results:**

We describe the case of a 35-year-old woman without relevant medical, toxicologic or psychiatric record that presents to our out-patient psychiatric clinic with post-traumatic stress disorder after five years of working as an internet content moderator and being exposed to visual traumatic content such as sexual assault and paedophilia. The clinical presentation consisted with one year of recurrent daily panic attacks, intrusive images about the traumatic exposure, intrusive thoughts, insomnia, vivid nightmares, avoidance of exposure to her son, distrust of the environment and intense fear for her son security. The disorder interfered in her capacity to work. The patient received psychological treatment and ISRS (Sertraline) was prescribed, however only partial response was reached with persistence of the majority of symptoms.

**Conclusions:**

The presented case suggests a temporal and symptom content relationship between the described work exposure and the appearance of emotional distress in a patient without PTSD history. Although previous evidence of secondary trauma in people exposed to indirect traumatic experiences has been reported, for example in healthcare professionals, the exposure to alien trauma through digital exposure as a workactivity is yet to be specifically examined. It is necessary to expand knowledge on the clinical expression of this phenomenon due to the observed recurrence of anxious and depressive symptomatology related to repeated exposure to traumatic content.

**Disclosure of Interest:**

None Declared

